# Inhibition of Activin A Signaling by Follistatin Alleviates Diabetic Kidney Disease Burden

**DOI:** 10.34067/KID.0000000877

**Published:** 2025-08-28

**Authors:** Érika Bevilaqua Rangel

**Affiliations:** Albert Einstein Research and Education Institute, Hospital Israelita Albert Einstein, São Paulo, Brazil and Nephrology Division, Federal University of São Paulo, São Paulo, Brazil

**Keywords:** chronic dialysis, CKD, diabetes mellitus, diabetic nephropathy, kidney transplantation

Diabetic kidney disease (DKD) is the leading cause of ESKD worldwide.^1^ It is a multifactorial disorder characterized by dysregulation of several biologic pathways, including inflammation, oxidative stress, cell death, autophagy, senescence, and fibrosis.^2^ These disruptions ultimately lead to structural and functional alterations in kidney compartments, contributing to the progression and burden of DKD.

Although lifestyle modifications and multitargeted therapies—including agents that address metabolic disturbances, hemodynamic changes, and inflammation, such as renin-angiotensin system blockers, sodium-glucose cotransporter 2 inhibitors, the mineralocorticoid receptor antagonist finerenone, and glucagon-like peptide-1 receptor agonists—are widely recommended,^3^ DKD frequently continues to progress. Thus, identifying novel therapeutic strategies to complement existing treatments is of paramount importance to halt DKD progression more effectively.

Senescence plays a central biologic role in aging, a known risk factor for chronic diseases such as cancer, cardiovascular and kidney diseases, diabetes mellitus, and neurodegenerative disorders.^4^ It is triggered by epigenetic factors, telomere attrition, DNA damage, and mitochondrial dysfunction, leading to the accumulation of reactive oxygen species. These alterations contribute to nutrient signaling dysfunction, chronic inflammation, stem cell exhaustion, and proteostatic imbalance, including the accumulation of misfolded or aggregated protein and impaired protein folding protein and degradation.

Two primary signaling pathways regulate senescence-induced cell arrest: the p16^INK4a^/Rb and p53/p21^CIP1^ pathway, both of which converge on the repression of cyclin-dependent kinase4/6. A hallmark of senescent cells is the senescence-associated secretory phenotype (SASP), which mediates many of their cell-extrinsic effects in an autocrine and paracrine. SASP promotes the recruitment and activation of both adaptive and innate immune cells, contributing to chronic low-grade inflammation—termed inflammaging—and tissue fibrosis.^4^ In addition, TGF-*β* can propagate the senescence phenotype to neighboring cells though paracrine signaling.

Within this context, activin A, a member of the TGF-*β* superfamily, has been implicated in multiple fibrotic processes, including epithelial-mesenchymal transition and fibroblast-myofibroblast transformation, leading to extracellular matrix deposition^5^ and the release of inflammatory cytokines.^6^ Circulating activin A has also been associated with senescence and SASP, contributing not only to aging but also to frailty and poor postsurgical outcomes.^7^

Consistent with these findings, Bian *et al.* reported elevated plasma levels of activin A in patients with DKD, which inversely correlated with eGFR.^8^ In addition, urinary activin A levels were associated with albuminuria, prompting further investigation into activin A as a therapeutic target for reducing inflammation, senescence, and fibrosis in DKD.

In this issue, Bian *et al.* evaluated the therapeutic potential of follistatin, a natural activin A antagonist, in C57BLKS/JLepr (db/db) mice treated with angiotensin II *via* subcutaneous osmotic mini pumps (1000 g/kg per minute).^9^ This well-established preclinical model of type 2 diabetes mellitus and DKD recapitulates key features of the disease, including glomerular and tubular injury and albuminuria. The authors also assessed the effects of follistatin on high glucose-treated human monocytes, renal fibroblasts, and renal tubular epithelial cells.

Follistatin is a secreted glycoprotein belonging to the secreted protein acidic and rich in cysteine, mainly secreted by mesenchymal stem cells.^10^ It modulates inflammation and immune responses, influencing cell survival, proliferation, differentiation, and migration. However, its role in kidney disease remains underexplored.

Bian *et al.* administered two intraperitoneal injections of 5 *µ*g of follistatin to db/db mice on days 15 and 18 after angiotensin II pump implantation, with euthanasia on day 28.^9^ Follistatin-treated mice exhibited reduced glomerular injury and lower albuminuria, while plasma creatinine and tubular injury were similar to untreated mice. Follistatin upregulated nephrin and Wilm's tumor 1 and downregulated kidney injury molecule-1 and Mac-2, suggesting preservation of podocyte markers and anti-inflammatory effects.

Furthermore, follistatin reduced circulating activin A levels and suppressed renal gene expression in diabetic mice treated with angiotensin II. It also decreased fibrotic markers (tenascin-C, collagen 1, and collagen 4), the senescent markers p19^CDKN2D^, and inflammatory markers (TNF-*α*, IL-1*β*, IL-6, and matrix metalloproteinase-2). Renal infiltration of inflammatory cells was also attenuated, with fewer F4/80-positive macrophages and downregulation of monocyte chemoattractant protein-1, toll-like receptor 4, NF kappa-light-chain-enhancer of activated B cells (NF-κB), CD45, and absent in melanoma 2 inflammasome. Follistatin also shifted the macrophage profile toward an anti-inflammatory phenotype by reducing M1 markers CD38 and CD86.

*In vitro*, follistatin (750 ng/ml) suppressed fibrotic activation in activin A (20 ng/ml)–treated renal fibroblasts, cultured under high-glucose conditions, as evidenced by reduced expression of *α*-smooth muscle actin, collagen 1, fibronectin, and collagen 4. Re-exposure to activin A reversed these effects, underscoring its profibrotic role.

In the phorbol-12-myristate-13-acetate–induced human macrophages (phorbol-12-myristate-13-acetate/U937) pretreated with high glucose and LPS (100 ng/ml), follistatin (500 or 750 ng/ml) treatment for 48 hours reduced the number of cells expressing NF-κB and downregulated gene expression of NF-κB, toll-like receptor 4, absent in melanoma 2, IL-1*β*, and IL-6, along with reduced Activin A secretion.

**Figure 1:**
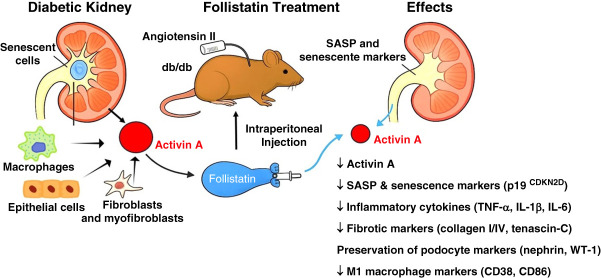
**Inhibition of activin A signaling by follistatin alleviates DKD burden.** CDK, cyclin-dependent kinase; DKD, diabetic kidney disease; SASP, senescence-associated secretory phenotype; WT-1, Wilm's tumor 1.

Collectively, these findings support the therapeutic potential of follistatin in DKD (Figure 1). Future preclinical studies should assess its long-term efficacy and explore its application in other CKD models, including acute-on-chronic kidney injury, either as monotherapy or in combination with other agents targeting inflammatory, senescence-related, and fibrotic signaling pathways. From a clinical perspective, monitoring activin A in response to pharmacologic and nonpharmacologic interventions could help establish its role as a biomarker for DKD progression and treatment response. Furthermore, clinical trials evaluating the safety and efficacy of follistatin in patients with DKD are warranted.
